# A198 THE LONG-TERM IMPACT OF ENVIRONMENTAL EXPOSURES ON HOST HEALTH AND THE RISK FACTORS OF CROHN'S DISEASE

**DOI:** 10.1093/jcag/gwac036.198

**Published:** 2023-03-07

**Authors:** M Xue, W Turpin, L Haim, S -H Lee, A Neustaeter, D Mei, W Xu, O Espin-Garcia, K L Madsen, D S Guttman, A M Griffiths, H Huynh, D Turner, R Panancionne, H Steinhart, G Aumais, A Bitton, K Jacobson, D Mack, K Croitoru

**Affiliations:** 1 Lunenfeld-Tanenbaum Research Institute; 2 Dalla Lana School of Public Health, University of Toronto, Toronto; 3 University of Alberta, Alberta; 4 Department of Cell & Systems Biology, University of Toronto; 5 Paediatrics, The Hospital for Sick Children, Toronto, Canada; 6 The Hebrew University of Jerusalem, Jerusalem, Israel; 7 University of Calgary, Calgary; 8 Montreal University; 9 McGill University, Montreal; 10 University of British Columbia, Vancouver; 11 Children’s Hospital of Eastern Ontario and University of Ottawa, Ottawa, Canada

## Abstract

**Background:**

Several environmental factors are associated with Crohn’s disease (CD) in large case-control studies; however, it is not clear how these factors maybe be influenced by age of exposure and if they are related to alterations in pre-disease biological markers of CD risk.

**Purpose:**

To investigate the association between environmental factors in different age groups with future risk of CD onset and assess their relation to other pre-disease biomarkers.

**Method:**

We used an environmental risk assessment questionnaire (ERA) to collect information from healthy first-degree relatives(FDR) of CD enrolled in the CCC-GEM project. ERA was a multi-item questionnaire querying 69 questions under 7 section headings: background, cultural/ethnic, smoking history, medical history, family history, environmental history and pet history. For the environmental and pet sections, current and historical (<1, 2-4, 5-15 years old) data was captured at the time of recruitment. We used Cox proportional hazard models to identify exposures associated with future CD onset. Next, we used regression models to identify the relationship of exposures with biological factors associated with CD risk previously identified by our group i.e.: i) intestinal permeability using urinary fractional excretion of lactulose to mannitol ratio (LMR) with LMR≥0.025 defined as abnormal; ii) subclinical inflammation using fecal calprotectin (FCP) with FCP≥100µg/g; and iii) fecal microbiome composition and diversity using 16S rDNA sequencing. Two-sided p<0.05 (or false discovery rate corrected p<0.05) were considered significant.

**Result(s):**

A total of 4289 FDRs were recruited, 47% were male, median recruitment age was 17.0 years[6-35]. After a median follow-up of 5.6-years (IQR=3.42-8.67), 86 FDRs developed CD. Living with a dog between age 5-15 (Hazard Ratio (HR)=0.61; 95% confidence interval (CI)=0.39-0.95), and a large family size (>3) in the first year of life (HR=0.41; 95% CI=0.22-0.89) were protective against CD onset. Conversely, having a bird at time of survey (HR=2.84; CI=1.37-5.90), and having a sibling with CD (HR=2.07; 95% CI=1.18-3.63) were risk factors for CD onset. We found that owning a dog between age of 5-15 (Odd Ratio(OR)=0.77, 95% CI=0.65-0.90) was significantly associated with LMR, nine taxa bacterial and higher chao1 diversity index. Having a bird at time of survey was significantly associated with FCP (OR=2.04, 95% CI=1.33-3.11). There was no association between large family size and having a CD sibling with gut microbiome, FCP or LMR.

**Conclusion(s):**

The study identified four environmental factors associated with future development of CD. Among them, exposure to dogs during early life was protective against CD onset and might be explained by its association with normal gut permeability and microbiome. We also identified that having a bird at recruitment increased risk of CD onset which might be mediated by an increase in subclinical inflammation.

**Submitted on behalf of the CCC-GEM consortium**

**Disclosure of Interest:**

None Declared

